# Genetic and molecular basis of floral induction in *Arabidopsis thaliana*

**DOI:** 10.1093/jxb/eraa057

**Published:** 2020-02-18

**Authors:** Atsuko Kinoshita, René Richter

**Affiliations:** 1 Department of Biological Sciences, Tokyo Metropolitan University, Tokyo, Japan; 2 School of Agriculture and Food, Faculty of Veterinary and Agricultural Sciences, The University of Melbourne, Melbourne, Australia; 3 Trinity College Dublin, Ireland

**Keywords:** Ageing pathway, epigenetics, gene regulatory networks, miRNAs, photoperiod, phytohormone, vernalization

## Abstract

Many plants synchronize their life cycles in response to changing seasons and initiate flowering under favourable environmental conditions to ensure reproductive success. To confer a robust seasonal response, plants use diverse genetic programmes that integrate environmental and endogenous cues and converge on central floral regulatory hubs. Technological advances have allowed us to understand these complex processes more completely. Here, we review recent progress in our understanding of genetic and molecular mechanisms that control flowering in *Arabidopsis thaliana*.

## Introduction

Flowering time control in plants is essential for their reproductive success and is also an important trait in agriculture. Plants have adapted several mechanisms to synchronize flowering so that they can maximize seed yields by carrying out fertilization and seed development at the optimal time (Purugganan and Fuller, 2009). In the model plant *Arabidopsis thaliana*, flowering is promoted by distinct environmental cues, such as daylength (photoperiod), winter (vernalization), and high ambient temperatures, as well as endogenous cues, such as plant age (ageing), the phytohormone gibberellin (GA), and the carbohydrate status (Ponnu *et al.*, 2011; Andrés and Coupland, 2012; Capovilla *et al.*, 2015). These signalling cues are perceived in the leaves and the shoot apical meristem (SAM) to induce flower formation. Over the last decades, extensive genetic studies have identified key regulators for flowering that function in the discrete flowering pathways (Koornneef *et al.*, 1998). Notably, these key regulators are encoded by transcription factors (TFs), cofactors for TFs, and chromatin remodellers. Furthermore, these genetic and epigenetic elements interact with each other to form a complex gene regulatory network (GRN).

In this review, we highlight the recent findings on photoperiod, age-related, and phytohormone-based mechanisms that sustain the plasticity in flowering time. This review is especially aimed to present a comprehensive summary of the recently characterized components that play important roles in the complex GRNs for flowering time control in Arabidopsis.

## Floral induction by the photoperiod pathway

Plants have evolved intricate mechanisms to measure fluctuations in daylength to accurately time the onset of flowering throughout seasonal progression, particularly at higher latitudes, and this phenomenon is known as photoperiodism (Garner and Allard, 1925). On the basis of their responses to photoperiod, plants are classified under three major groups: short-day (SD) plants initiate flowering when the night exceeds a critical length (normally in autumn); long-day (LD) plants flower when the night falls below a critical length (normally in late spring and summer); and day-neutral plants flower after attaining a certain developmental stage independently of daylength (Andrés and Coupland, 2012).

### Regulatory network of long-day signals in the model plant Arabidopsis

Arabidopsis late flowering time mutants were initially isolated based on their increased total number of leaves (Rédei, 1962; Koornneef *et al.*, 1991). Genes that have been isolated from these screens are key regulators in the process of floral induction in LDs, such as *FLAVIN-BINDING, KELCH REPEAT, F-BOX1* (*FKF1*), *GIGANTEA* (*GI*), *CRYPTOCHROME2* (*CRY2*), *FLOWERING LOCUS E* (*FE)*, *CONSTANS* (*CO*), and *FLOWERING LOCUS T* (*FT*) (Andrés and Coupland, 2012; Song *et al.*, 2015). Photoperiodic perception occurs in leaves, a tissue where these genes are expressed (Takada and Goto, 2003; An *et al.*, 2004; Wigge *et al.*, 2005). Although *FKF1* and *GI* display a broad expression pattern, they overlap with that of *CO* and *FT* in the vascular tissue of leaves (Song *et al.*, 2013).

### Molecular basis of long-day-dependent transcriptional activation of *CONSTANS*

**Fig. 1.**
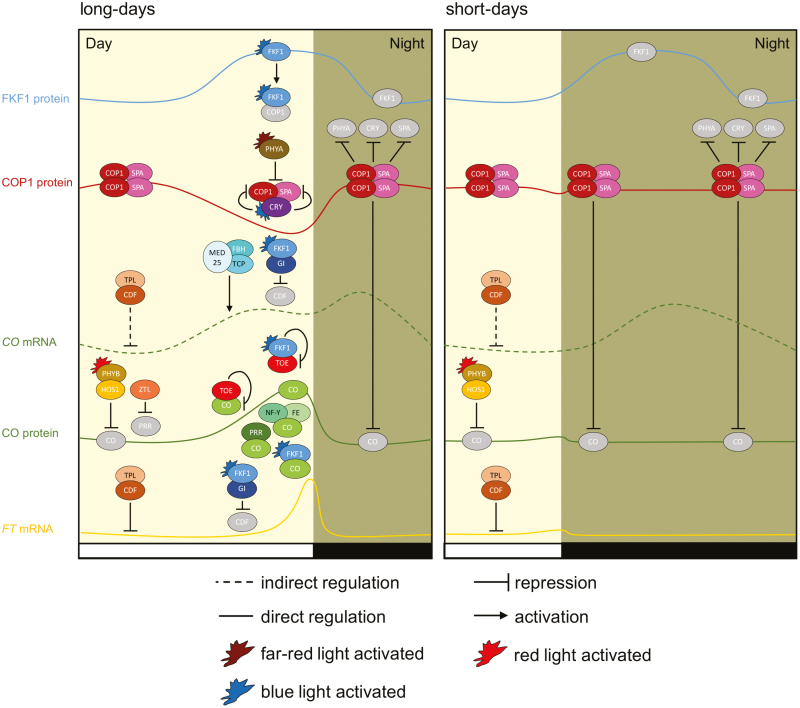
CONSTANS (CO) controls photoperiodic flowering of Arabidopsis. Left: *CO* mRNA peaks 12–16 h after dawn in the light under LD conditions and induces floral transition through the activation of *FLOWERING LOCUS T* (*FT*) in Arabidopsis. Right: *CO* mRNA peaks in the dark under short-day conditions and the CO protein is targeted for proteasomal degradation through the activity of the COP1–SPA ubiquitin ligase complex. In the morning, CO protein is degraded by the PHYB pathway.

LD-dependent flowering is associated with the activation of the photoperiodic pathway through the transcriptional regulator CO, a member of the B-box (BBX) zinc family which contains two N-terminal B-boxes and a C-terminal CONSTANS, CONSTANS-LIKE, TIMING OF CAB EXPRESSION1 (TOC1) (CCT) DNA-binding domain (Fig. 1) (Strayer *et al.*, 2000; Robson *et al.*, 2001; Khanna *et al.*, 2009; Gangappa and Botto, 2014).

Transcriptional activation of *CO* is light dependent and controlled through the formation of a complex between the ubiquitin ligase FKF1 and GI in late afternoon (regarded as external coincidence) (Mizoguchi *et al.*, 2005; Sawa *et al.*, 2007, 2008). Although the circadian clock-regulated genes *FKF1* and *GI* have differently entrained expression rhythms depending on daylength, they have the same phase in LDs (regarded as internal coincidence) but not in SDs (Sawa *et al.*, 2008). GI protein accumulates in late afternoon and stabilizes FKF1 in a circadian manner to target its substrate CYCLING DOF FACTORs (CDFs) for proteasomal degradation (Fowler *et al.*, 1999; Park *et al.*, 1999; Fornara *et al.*, 2009). CDFs contribute to the correct interpretation of the seasonal information by forming a repressor complex with TOPLESS (TPL) (Liu and Karmarkar, 2008; Goralogia *et al.*, 2017). The rhythmic light-controlled turnover of CDFs releases the transcriptional repression on *CO* which peaks in its expression at dusk (Imaizumi *et al.*, 2005; Fornara *et al.*, 2009). The vascular-expressed and photoperiod-specific FLOWERING BHLH (FBH) proteins form an activator complex with the otherwise miRNA319 (miR319)-sensitive TEOSINTE BRANCHED/CYCLOIDEA/PCF (TCP) TFs and bind to a *CO* proximal promoter region (Palatnik *et al.*, 2003; Ito *et al.*, 2012; Kubota *et al.*, 2017; Liu *et al.*, 2017). PHYTOCHROME AND FLOWERING TIME1/MEDIATOR25 (PFT1/MED25), a Mediator complex component required to orchestrate RNA polymerase II-dependent transcription, conveys regulatory information from the FBH–TCP complex to activate photoperiodic expression of *CO* in LDs (Cerdán and Chory, 2003; Iñigo *et al.*, 2012; Ito *et al.*, 2012; Liu *et al.*, 2017). However, it is of major interest to explore the genetic interaction between FBHs and TCPs in the regulation of *CO* expression since both transcriptional activators may function cooperatively and/or independently.

### Molecular mechanisms regulating CONSTANS protein stability and function

Post-translational control of CO protein is an important determinant for floral induction in response to LDs. The phosphorylated form of the CO protein is preferentially degraded in the dark by the 26S proteasome through the activity of the E3 ubiquitin ligase complex CONSTITUTIVE PHOTOMORPHOGENIC 1 (COP1) and SUPPRESSSOR OF PHYTOCHROME A-105 (SPA) (Hoecker *et al.*, 1998, 1999; Laubinger *et al.*, 2006; Jang *et al.*, 2008; Liu *et al.*, 2008; Sarid-Krebs *et al.*, 2015). While light-activated FKF1 conveys daylength-dependent transcriptional activation of *CO* and *FT*, FKF1 also increases the protein level of CO by inhibiting functional COP1 homodimerization (Song *et al.*, 2012; Lee *et al.*, 2017). In addition, CO protein stability is increased through a blue-light-dependent binding to FKF1 (Nelson *et al.*, 2000; Demarsy and Fankhauser, 2009; Song *et al.*, 2012). The blue light photoreceptors CRY1 and CRY2 enhance CO protein stability through sequestration of SPA1 from the COP1–SPA1 complex, whereas the CRY2–COP1 interaction reduces COP1–SPA catalytic activity under blue light (Liu *et al.*, 2008; Lian *et al.*, 2011; Zuo *et al.*, 2011; Holtkotte *et al.*, 2017). On the other hand, COP1 and SPA proteins most probably contribute to the blue-light-dependent proteasomal degradation of CRY2 (Shalitin *et al.*, 2002; Liu *et al.*, 2016). Similarly, far-red light activation of the phytochrome A (phyA) photoreceptor directly disrupts SPA1–COP1 interaction in the late afternoon, whereas the red/far-red light photoreceptor phytochrome B (phyB) facilitates CO protein degradation in the morning (Valverde *et al.*, 2004; Sheerin *et al.*, 2015). An attenuation of the phyA-dependent inhibition of the COP1–SPA complex is mediated through a COP1-dependent proteolysis of phyA, thereby creating an autoregulatory feedback loop on COP1 E3 ubiquitin ligase function (Seo *et al.*, 2004). Likewise, a light-dependent (auto)-ubiquitylation pathway for the COP1–SPA2 complex has been proposed, where COP1 mediates ubiquitylation and degradation of SPA2 (Chen *et al.*, 2015).

Alternative splicing of *CO* mRNA produces the CCT-truncated variant COβ that promotes HIGH EXPRESSION OF OSMOTICALLY RESPONSIVE GENE 1 (HOS1), a RING-finger-containing E3 ubiquitin ligase, and COP1-dependent proteasomal turnover of the full-length protein COα, whereas COβ is resistant to the activity of these E3 ubiquitin ligases (Gil *et al.*, 2017). The HOS1-mediated reduction in COα protein depends on phyB in the morning (Lazaro *et al.*, 2012, 2015). Plants overexpressing COβ are strongly delayed in flowering, which is due to a loss of interaction between COα and the CO-stabilizing protein FKF1 on one hand and the inhibition of COα-NUCLEAR FACTOR-Y (NF-Y) complex formation on the other hand (Wenkel *et al.*, 2006; Gil *et al.*, 2017).

The destabilization of CO protein in the morning is attenuated through the formation of a complex with PSEUDO RESPONSE REGULATOR9 (PRR9), a central component of the circadian clock, whereas the related family members TOC1/PRR1, PRR5, and PRR7 engage in interactions with CO mainly in the late afternoon (Strayer *et al.*, 2000; Farré and Liu, 2013; Hayama *et al.*, 2017). PRRs repress *CDF1* transcription, thus allowing *CO* mRNA to rise in the late afternoon (Nakamichi *et al.*, 2007). In addition to its main function as an E3 ubiquitin ligase to control proteasomal degradation of central clock proteins TOC1 and PRR5, ZEITLUPE (ZTL) enhances destabilization of CO protein in the morning and changes intracellular localization of FKF1 in the late afternoon (Somers *et al.*, 2000; Más *et al.*, 2003; Han *et al.*, 2004; Kiba *et al.*, 2007; Takase *et al.*, 2011; Song *et al.*, 2014). Thus, it is imperative to understand in detail how PRRs may function to reduce COP1 activity on CO during the day and whether PRRs might also bind to the *FT* promoter.

### Integration of floral transition signals at *FLOWERING LOCUS T*

As a consequence of the transcriptional and post-translational regulation, CO protein peaks at late afternoon in LDs. CO binds to a proximal CO response element (CORE) in the promoter of *FT*, and interacts with the NF-Y–FE complex that binds to the distal enhancer element in the *FT* promoter, to induce DNA looping at *FT* and to sustain enhanced transcriptional activation of *FT* in late afternoon (Fig. 1) (Ben-Naim *et al.*, 2006; Wenkel *et al.*, 2006; Adrian *et al.*, 2010; Song *et al.*, 2012; Cao *et al.*, 2014; Gnesutta *et al.*, 2017; Hayama *et al.*, 2017; Shibuta and Abe, 2017). A recent study identified another crucial enhancer with additive effects on flowering time in inductive conditions that is located downstream of *FT* and most probably contributes to photoperiod-dependent activity (Zicola *et al.*, 2019).

**Fig. 2.**
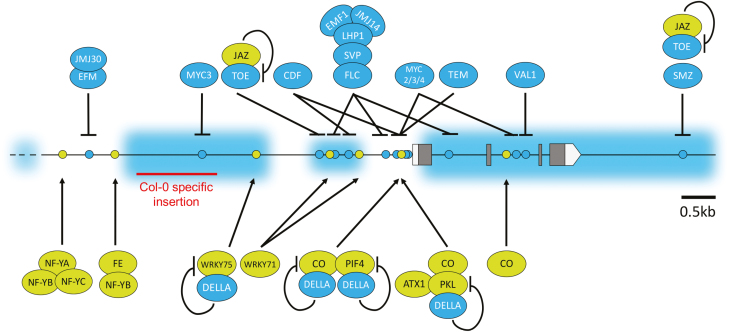
*FLOWERING LOCUS T* (*FT*) integrates seasonal cues through the tight control of floral activators and repressors. The balance between transcriptional activators and repressors determines the transcriptional status of *FT*. Gene model of *FT* depicting the 5'- and 3'-untranslated regions (light grey boxes) and exons (dark grey boxes). The cognate DNA-binding sites for the transcriptional regulators of *FT* are depicted by colour-coded circles (green, active; cyan blue, repressive). Transcriptional activators and repressors are depicted in green and cyan blue, respectively. The repressive epigenetic H3K27me3 marks at *FT* are highlighted by the light blue cloud.

In addition to the photoperiod-specific *FT* regulation, several mechanisms regulate proper timing of flowering, most probably by maintaining the intricate balance between floral repressors and activators (Fig. 2). The two functionally redundant genes *TEMPRANILLO1* (*TEM1*) and *TEM2* act in the early developmental stage to block floral transition. Thus, an important mechanism for *FT* regulation is the balance between *CO* and *TEM* genes (Castillejo and Pelaz, 2008). Both TEM1 and TEM2 directly bind to *FT*, whereas TEM2 shows a specific binding to the *FT* homologue *TWIN SISTER OF FT* (*TSF*) under low ambient temperatures (Yamaguchi *et al.*, 2005; Castillejo and Pelaz, 2008; Marín-González *et al.*, 2015).

A morning-specific inhibition of CO function occurs through an interaction with the miR172-sensitive APETALA2 (AP2)-type transcriptional regulator TARGET OF EAT1 (TOE1), whereas FKF1 relieves this repressive constraint by binding TOE1 (Zhang *et al.*, 2015). Other miR172-sensitive subfamilies of AP2-like transcriptional regulators, including *AP2*, *TOE2*, *TOE3*, *SCHNARCHZAPFEN* (*SNZ*), and *SCHLAFMÜTZE* (*SMZ*), also contribute to the repression of flowering under inductive and non-inductive photoperiod conditions (Schmid *et al.*, 2003; Yant *et al.*, 2010). However, a direct binding to a region downstream of *FT* was shown only in plants overexpressing SMZ or TOE1 (Mathieu *et al.*, 2009; Zhai *et al.*, 2015).

The major advances in the understanding of the complex GRNs contributing to *FT* activation were made over the last years under standard laboratory growth conditions. Interestingly, a recent report showed that the *FT* expression is actually induced not only in the evening but also in the morning under natural LD conditions. The morning-specific increases in CO protein stability and *FT* transcript level were reproduced under refined laboratory conditions, in which the ratio of far-red light to red light and the daily temperature are modified (Song *et al*., 2018). Thus, recreating natural plant growth conditions in laboratories will help to identify previously uncharacterized mechanisms contributing to floral induction.

### Epigenetic regulation of *FLOWERING LOCUS T*

Epigenomic modifications are important for a widespread set of biological and developmental processes in higher eukaryotes. Epigenetic information involves covalent modifications of chromosomal histones that translate into changes in chromatin structure and are associated with either gene repression or activation (Steffen and Ringrose, 2014). In Arabidopsis, *FT* is a target of the Polycomb repressive complex 2 (PRC2) component CURLY LEAF (CLF), a methyltransferase that catalyses the deposition of histone H3 lysine 27 tri-methylation (H3K27me3), one of the repressive marks, and is associated with gene silencing (Fig. 2) (Goodrich *et al.*, 1997; Jiang *et al.*, 2008; Lopez-Vernaza *et al.*, 2012). The B3-domain-containing TF VIVIPAROUS1/ABSCISIC ACID INSENSITIVE3-LIKE1 (VAL1) binds to two intronic RY (purine and pyrimidine nucleotides) motifs in *FT* and orchestrates recruitment of PRC components before dusk to mediate H3K27me3 deposition on *FT* chromatin (Reidt *et al.*, 2000; Jia *et al.*, 2014; Luo *et al.*, 2018; Jing *et al.*, 2019*a*). Epigenetic silencing of *FT* is sustained by the activity of LIKE HETEROCHROMATIN PROTEIN1 (LHP1) which binds to H3K27me3 sites in *FT* through its chromodomain (Gaudin *et al.*, 2001; Turck *et al.*, 2007; Zhang *et al.*, 2007; Exner *et al.*, 2009; Adrian *et al.*, 2010). In contrast, formation of NF-YB-YC-CO complexes antagonizes CLF binding and deposition of H3K27me3 at *FT* (Takada and Goto, 2003; Liu *et al.*, 2018; Luo *et al.*, 2018). Similarly, binding of the PRC1 component EMBRYONIC FLOWER1 (EMF1) to *FT* is disrupted by the photoperiodic activity of CO, thus resulting in the activation of *FT* (Sung *et al.*, 1992; Calonje *et al.*, 2008). A physical interaction between CO and the CHD3 chromatin-remodelling factor PICKLE (PKL) enhances the binding of both regulators to *FT* chromatin and thus promotes floral transition (Ogas *et al.*, 1997, 1999; Jing *et al.*, 2019*c*). Although genome-wide studies demonstrate that PKL predominantly co-localizes with the repressive epigenetic mark H3K27me3, PKL was also found to be associated with gene activation (Zhang *et al.*, 2008, 2012; Jing *et al.*, 2013; Zhang *et al.*, 2014). A recent study suggested that PKL might act as a pre-nucleosome maturation factor and promotes retention of epigenetic marks after DNA replication and/or transcription, which can provide a plausible explanation for its dual role as activator and repressor in gene transcription (Carter *et al.*, 2018). PKL also contributes to the relaxation of chromatin at *FT* through the formation of a complex with the H3K4me2/3-specific methyltransferase ARABIDOPSIS HOMOLOG OF TRITHORAX1 (ATX1), thus preventing PcG-mediated silencing of *FT* (Jing *et al.*, 2019b).

Overexpression of RELATIVE OF EARLY FLOWERING 6 (REF6), a Jumonji (JMJ) domain-containing histone H3K27me3 demethylase, activates transcription of *FT* (Noh *et al.*, 2004; Lu *et al.*, 2011). Conversely, *ref6* mutants are late flowering and this phenotype can be attributed to the derepression of the floral repressor *FLOWERING LOCUS C* (*FLC*) (Noh *et al.*, 2004). *REF6* and the homologous genes *EARLY FLOWERING 6* (*ELF6*) and *JMJ13* have redundant functions; however, *REF6* plays the major role in shaping the genome-wide distribution of H3K27me3 (Yan *et al.*, 2018).

Genome-wide studies have revealed that in Arabidopsis, genes with H3K27me3 signatures are often decorated with the active chromatin mark histone H3 lysine 4 di-methylation (H3K4me2) in a mutually exclusive manner (Zhang *et al.*, 2009; Engelhorn *et al.*, 2017). Polycomb-mediated gene repression of *FT* is linked to the EMF1-interacting H3K4me2-specific demethylases JMJ14, JMJ15, and JMJ18 (Lu *et al.*, 2010; Yang *et al.*, 2012*a*, *b*). The homologous plant-unique bivalent Bromo adjacent homology (BAH)-plant homeodomain (PHD) finger domain-containing proteins EARLY BOLTING IN SHORT DAY (EBS) and SHORT LIFE (SHL) prevent premature flowering through a mechanism which involves binding to PRC1 complex components to further sustain Polycomb-mediated gene silencing of *FT* (Piñeiro *et al.*, 2003; López-González *et al.*, 2014; Li *et al.*, 2018). Although EBS and SHL have been characterized as bivalent readers capable of switching their binding preference between H3K4me3- and H3K27me3-marked chromatin, a hypothesized signal that triggers this switch still awaits its identification.

The histone modification H3K36me3 marks transcriptionally active genes and has key roles in the regulation of splicing (Pajoro *et al.*, 2017). Genome-wide studies in Arabidopsis and maize indicated that H3K36me3 is distributed across gene bodies with major abundance at the 5' region, which is significantly different from the H3K36me3 distribution pattern in mammals (He *et al.*, 2013; Li *et al.*, 2015). Although *FT* is a target of H3K36me3 modification, little is known about the mechanism for establishing H3K36me3 at *FT*. However, a recent report shows that the H3K36me3-specific histone demethylase JMJ30 is recruited by the MYB-type TF EARLY FLOWERING MYB PROTEIN (EFM), which binds to a distal site in the *FT* promoter, to catalyse the removal of H3K36me2/3 at *FT* and thus regulates the proper timing for reproduction (Yan *et al.*, 2014).

### Nucleosomal organization contributes to *FLOWERING LOCUS T* regulation

Nucleosome organization and distribution contribute to a tight control over gene transcription. Genome-wide studies have indicated that different levels of the histone variant H2A.Z along the genes contribute to the regulation of gene activity (To and Kim, 2014). Eviction of H2A.Z-containing nucleosomes is crucial for PHYTOCHROME INTERACTING FACTOR 4- (PIF4) induced *FT* activation at high ambient temperatures (Kumar *et al.*, 2012; Gómez-Zambrano *et al.*, 2018). Notably, a thermosensory function has been assigned to phyB, thus translating temperature and light effects into targeted degradation of PIF proteins (Jung *et al.*, 2016*a*; Legris *et al.*, 2016). Although rather speculative, these findings imply a possible scenario in which phyB modulates the floral response under changing environmental conditions. Moreover, the photoperiodic, thermosensory, and GA pathways converge on the CO–PIF4/5–DELLA module to promote flowering at high temperatures in SDs (Galvão *et al.*, 2015; Fernández *et al.*, 2016). Sliding and eviction of nucleosomes are promoted by BRAHMA (BRM), a member of SWI2/SNF2 chromatin remodelling ATPases (Farrona *et al.*, 2007; Ojolo *et al.*, 2018). BRM regulates flowering time through transcriptional repression of *FT* in LDs (Farrona *et al.*, 2004, 2011). Notably, H2A.Z and BRM cooperate in the control of *FT* transcription, which is further supported by a recent report that shows context-dependent regulatory roles of BRM and H2A.Z (Torres and Deal, 2019).

### Natural variation at *FLOWERING LOCUS T*

Although chromatin remodellers facilitate chromatin opening, they have less effect on the binding specificity of TFs. Nevertheless, promoter and *cis*-regulatory variation are instrumental for gene regulation since they contribute to changes in TF binding and chromatin structure (de Meaux, 2018). An Arabidopsis accession Col-0-specific insertion (Block ID) in *FT* was identified and shown to contribute to photoperiodic regulation of *FT* (Adrian *et al.*, 2010; Bao *et al.*, 2019). In more detail, large insertions–deletions (INDELs) overlapping with Block ID correlated with geographical clines which are widespread and account for natural variation at *FT* (Liu *et al.*, 2014). Likewise, CO-associated flowering time diversity was shown to be linked to natural variation in *cis*-regulatory sequences of the *CO* promoter (Rosas *et al.*, 2014). As for *FT*, Liu (2014) suggested that *cis*-regulatory variation could be adaptive by conferring differences in the control of *FT* which translates into increased fitness (Schwartz *et al.*, 2009; Liu *et al.*, 2014). *Cis*-regulatory changes in the MYC3-binding site at *FT* to suppress its activation under non-inductive SD conditions is an elementary pillar of natural variation in the control of photoperiodic flowering responses (Bao *et al.*, 2019). Targeted DNA methylation of *cis*-regulatory elements and intronic regions in *FT* helped to further unveil additional *cis*-regulatory elements with functional roles in the regulation of *FT* in the photoperiodic response pathway (Deng and Chua, 2015; Zicola *et al.*, 2019). It is noteworthy that these sites are involved in the targeted recruitment of PIF4/5 and the floral repressors FLC, FLOWERING LOCUS M (FLM), and VAL1 (Searle *et al.*, 2006; Gu *et al.*, 2013; Lee *et al.*, 2013; Pedmale *et al.*, 2016; Jing *et al.*, 2019*a*).

### FT, a leaf-derived systemic signal that moves to the shoot apical meristem

The concept of florigen was first proposed in the 1930s as a graft-transmissible leaf-derived florigenic signal that is responsive to photoperiodic stimuli and induces floral initiation at the SAM (Chailakhyan, 1936). By virtue of genetic and molecular experiments in *Arabidopsis thaliana* and rice in the past two decades, the FT protein has been characterized as the long-sought florigen (Corbesier *et al.*, 2007; Jaeger and Wigge, 2007; Mathieu *et al.*, 2007; Tamaki *et al.*, 2007). FT shares homology with phosphatidylethanolamine-binding proteins (PEBPs) or RAF kinase inhibitor proteins (RKIPs), and its ligand-binding domain is evolutionarily conserved from bacteria to mammals and plants (Kardailsky *et al.*, 1999; Kobayashi *et al.*, 1999). FT protein is expressed in the phloem companion cells of the leaves and is shown to diffuse in the SAM to induce flowering, which indeed fits with the concept of florigen (Corbesier *et al.*, 2007; Jaeger and Wigge, 2007; Mathieu *et al.*, 2007; Tamaki *et al.*, 2007). A recent report further confirmed the transport of FT protein from leaves to the SAM, by combining an improved bimolecular fluorescence complementation (iBiFC) assay and a heat shock-inducible gene expression system (Abe *et al.*, 2019). FT protein levels gradually decrease once floral transition occurs, although *FT* mRNA is still transcribed with its typical peak in expression at dusk, and this post-translational control is mediated by proteases which cleave the C-terminal part of FT (Kim *et al*., 2016). Trafficking of FT to the vegetative SAM depends on the endoplasmic reticulum (ER) membrane protein FT-INTERACTING PROTEIN 1 (FTIP1), a member of the family of multiple C2 domain and transmembrane region proteins (MCTPs), which facilitates the export of FT from phloem companion cells (CCs) to sieve elements (SEs) (Liu *et al.*, 2012). The plasma membrane-resident syntaxin-like Q-SNARE, SYNTAXIN OF PLANTS 121 (SYP121), interacts with QUIRKY (QKY/MCTP15) to regulate FT movement to the plasmalemma in CCs through the endosomal trafficking pathway (Liu *et al.*, 2019). The long-distance transport of FT from leaves to the SAM through the phloem stream is facilitated by the heavy metal-associated (HMA) domain-containing protein SODIUM POTASSIUM ROOT DEFECTIVE 1 (NaKR1), which is activated by CO and FE in leaf vascular tissue and shown to interact with FT (Zhu *et al.*, 2016; Shibuta and Abe, 2017). Nevertheless, uploading of FT to the phloem and unloading in the SAM are actively regulated processes, at least in cucurbit plants. Furthermore, trafficking of FT is strongly influenced by phloem fluxes and concentrations of major sugars in phloem sap as they exhibit diurnal and developmental changes (Mitchell *et al.*, 1992; Savage *et al.*, 2013; Yoo *et al.*, 2013).

### Formation of the florigen activation complex

Transport of FT from leaves to the vegetative SAM induces floral transition which is characterized by morphological changes and rewiring of transcriptional networks that culminate in floral induction (Jacqmard *et al.*, 2003; Torti *et al.*, 2012). The basic leucine zipper (bZIP) domain TF FD is expressed in the SAM and forms a transient complex with FT/TSF to induce floral meristem identity genes such as *APETALA1* (*AP1*) (Abe *et al.*, 2005, 2019; Wigge *et al.*, 2005). This interaction is indirect since the 14-3-3 protein GF14c bridges the interaction between HEADING DATE 3A (HD3A), the rice orthologue of FT, and rice OsFD1 (Taoka *et al.*, 2011). Phosphorylation of FD by the SAM-expressed CALCIUM-DEPENDENT PROTEIN KINASE 6 (CDPK6) and CDPK33 promotes florigen activation complex (FAC) formation to coordinate floral transition (Kawamoto *et al.*, 2015; Collani *et al.*, 2019). In contrast, the *FT*-related gene *TERMINAL FLOWER1* (*TFL1*), which is a key floral repressor, interacts with the unphosphorylated form of FD via 14-3-3 proteins. Moreover, it has been suggested that the transcriptionally inactive ternary FD–14-3-3–TFL1 complex represents the ground state at the SAM (Collani *et al.*, 2019). As TFL1 acts through FD, TFL1 counterbalances incoming FT signals to maintain the centre of the SAM in a vegetative state through an interlocking feedback loop (Kobayashi *et al.*, 1999; Hanano and Goto, 2011; Jaeger *et al.*, 2013; Lee *et al.*, 2019). Modulation of FAC activity also occurs through the specific binding of FT to diurnally changing molecular species of phosphatidylcholine (PC) (Nakamura *et al.*, 2014). Lipid binding seems to be important for FT function, as several loss-of-function *ft* alleles carry point mutations within the ligand-binding pocket (Kobayashi *et al.*, 1999). Although FT and TSF are not required for FD binding to DNA, their presence increase the enrichment of FD to a subset of genes that regulate flowering time and floral organ identity (Collani *et al.*, 2019).

### Modulation of the floral response through integration of transcription factors with the FT–FD module

A recent work has shed light on the importance of the FD–FT protein interaction network and how this relates to the associated transcriptional output (Li *et al.*, 2019). FD was found to interact with class II CINCINNATA (CIN)-like TCP5, TCP13, and TCP17, which facilitate the DNA binding of FD to the floral meristem identity gene *AP1* (Martín-Trillo and Cubas, 2010; Li *et al.*, 2019). This study concluded that the class II CIN-like TCPs and FD synergistically activate downstream signalling (Li *et al.*, 2019). Similarly, the age-related miR156-sensitive SQUAMOSA PROMOTER-BINDING PROTEIN (SBP)-LIKE (SPL) TFs SPL3, SPL4, and SPL5 hijack the FD–FT signalling module through physical interaction with FD to enhance its DNA binding and to synergistically activate *AP1* expression (Jung *et al.*, 2016*b*). It is noteworthy that *SPL3* and *FT* mutually cross-activate each other, thereby creating a coherent feedforward loop (Alon, 2007; Jung *et al.*, 2012; Kim *et al.*, 2012; Lee *et al.*, 2012). Nevertheless, regardless of which protein complexes assemble at *AP1* and how they modulate the binding behaviour of FD, a consensus is that these proteins synergistically activate the expression of *AP1*. Of note, SPL9 was also shown to bind to *AP1* to trigger the onset of flower formation and interacts with the mir319-sensitive TCP4 to regulate leaf complexity. It is thus speculated whether TCPs and SPLs may cooperate to facilitate recruitment of the FD–FT module (Rubio-Somoza *et al.*, 2014; Yamaguchi *et al.*, 2014). On the contrary, the class II TCP family member BRANCHED1 (BRC1)/TCP18 protein interacts with FT and TSF to repress premature floral transition of axillary meristems through modulation of florigen activity in the axillary buds, demonstrating multifaceted roles and interaction potentials for TCPs (Niwa *et al.*, 2013).

## Age-related floral induction under non-inductive conditions

**Fig. 3.**
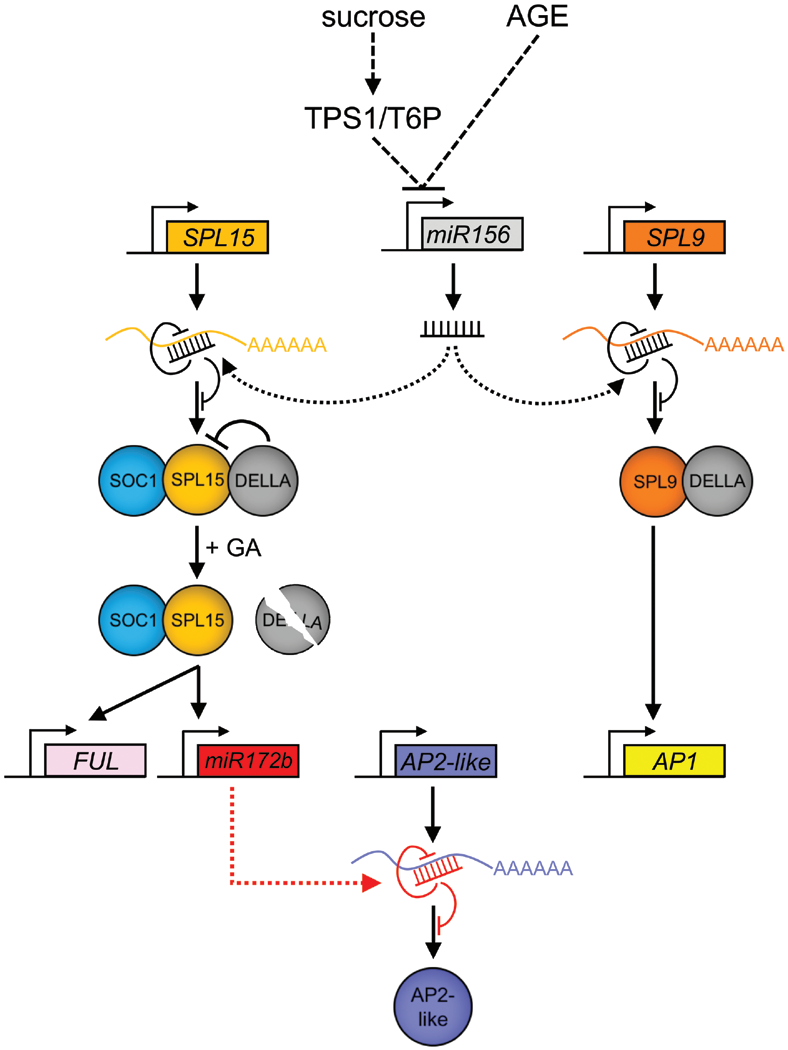
The age-related transcriptional network contributes to the floral transition at the shoot apical meristem (SAM). Sugars and the plant age reduce *miR156* levels at the SAM. As a consequence, transcript and protein levels of SQUAMOSA PROMOTER-BINDING PROTEIN (SBP)-LIKEs (SPLs) increase. Gibberellins (GAs) promote DELLA protein degradation, the latter of which interacts with SPL15 to inhibit its function. In contrast, DELLAs enhance SPL9-dependent transcriptional activation of *APETALA1* (*AP1*). SUPPRESSOR OF OVEREXPRESSION OF CONSTANS 1 (SOC1) cooperates with SPL15 to induce expression of *miR172b* and *FRUITFULL* (*FUL*). As a result, *mir172b* inactivates transcripts of the AP2-like floral repressor genes.

Before plants become competent to flower and reproduce, the shoot has to undergo the phase of vegetative growth, which can be further divided into the juvenile and the adult vegetative phase. These phases are accompanied by changes in growth pattern and body forms, and increases in photosynthetic capacity, which are particularly recognizable in perennials rather than in annual species such as Arabidopsis. During the transition from the juvenile to adult phase also known as vegetative phase change, plants acquire reproductive competence. Eventually, the reproductive phase change is characterized by the switch from vegetative to reproductive growth, a process in which the SAM adopts an inflorescence meristem identity. It has become increasingly clear in recent years that the juvenile to adult phase and reproductive phase use similar molecular and genetic mechanisms. In particular, the miR156–SPL and miR172–AP2 modules are likely to be the central regulatory hubs and required to coordinate the transitions of the discrete phases in a timely manner (Fig. 3) (Huijser and Schmid, 2011; Hyun *et al.*, 2017).

### Age-related decline in miR156

Floral induction under non-inductive SD conditions requires the activity of the phytohormone GA and the age-dependent reduction in the levels of miR156, which is one of the most abundant miRNAs in Arabidopsis with the highest levels at the seedling stage (Wilson *et al.*, 1992; Axtell and Bartel, 2005; Schwab *et al.*, 2005). miR156 and miR157, which are encoded by eight and four precursors, respectively, repress *SPL* gene expression in a threshold-dependent manner (Rhoades *et al.*, 2002; He *et al.*, 2018). Although miR157 is more abundant than miR156, the major role in the regulation of vegetative phase can be attributed to miR156, which is also one of the most conserved miRNAs among various plant species (Zhang *et al.*, 2006; Yang *et al.*, 2011; He *et al.*, 2018). A recent report hypothesized that miR156 diffuses non-cell autonomously from the SAM into leaf primordia to promote juvenile leaf identity (Fouracre and Poethig, 2019). In further support of this notion, previous studies found that miR156 acts as a mobile signal in potato and maize (Poethig, 1988; Dudley and Poethig, 1993; Bhogale *et al.*, 2014). Following the juvenile growth, miR156 is expressed in leaves and increased in abundance as leaves expand (Fouracre and Poethig, 2019). To confer a gradual transition from the juvenile to adult phase, miR156 progressively declines in successively developing shoot-derived leaf primordia (He *et al.*, 2018). The signalling activity of HEXOKINASE1 (HXK1) and sugar, which acts as a mobile signal, contributes to the reduction in miR156 abundance (Yang *et al.*, 2013; Yu *et al.*, 2013; Buendia-Monreal and Gillmor, 2017). Furthermore, *TREHALOSE-6-PHOSPHATE (T6P) SYNTHASE 1* (*TPS1*) and T6P, which has been suggested to function as a signalling molecule of sugar status in plants, are also likely to contribute to the reduction in miR156 abundance (Lunn *et al.*, 2006; Wahl *et al.*, 2013). In addition, *tps1* mutants are extremely late flowering even in LDs, and disable to induce oscillating *FT* expression during a day (Wahl *et al.*, 2013).

### Epigenetic and transcriptional regulation of MIR156

The transcription of *MIR156a/c* is repressed at the adult phase by epigenetic regulators such as BMI1, VAL1/2, CLF, and its homologue SWINGER (SWN), while BRM antagonizes mainly the function of SWN at the juvenile phase (Picó *et al*., 2015; M. Xu *et al.*, 2016*a*; Y. Xu *et al.*, 2016; Merini *et al.*, 2017). The ATP-dependent SWR1 chromatin remodelling complex (SWR1-C) contributes to nucleosomal dynamics at *MIR156a/c*, while ACTIN-RELATED PROTEIN6 (ARP6) promotes H2A.Z incorporation to facilitate ARABIDOPSIS TRITHORAX-RELATED7 (ATXR7)-dependent active chromatin formation at *MIR156a/c* (Tamada *et al.*, 2009; Choi *et al.*, 2016; Xu *et al.*, 2018).

### SPLs induce developmental transitions

Two important developmental transitions—the juvenile to adult transition and the vegetative to reproductive transition—in Arabidopsis are controlled through miR156-targeted inactivation of *SPL* mRNAs by cleavage and translational inhibition (Schwab *et al.*, 2005; Gandikota *et al.*, 2007; Hyun *et al.*, 2017). The SPL family is comprised of 16 genes in Arabidopsis that are divided into two groups (Guo *et al.*, 2008; Xing *et al.*, 2010). miR156 recognition sites were reported for 11 members of these *SPL* genes. Among them, *SPL2*, *SPL9*, *SPL10*, *SPL11*, *SPL13*, and *SPL15* were shown to be strongly associated with floral transition, whereas *SPL3*, *SPL4*, and *SPL5* promote floral meristem identity (Schwarz *et al.*, 2008; Wang *et al.*, 2009; Wu *et al.*, 2009; Yamaguchi *et al.*, 2009; Hyun *et al.*, 2016; M. Xu *et al.*, 2016*b*).

SPL9 and SPL15 bind to the promoter of the miR172b gene to promote its expression, which is required to inactivate transcripts of floral repressor genes of the AP2-like family (Wu *et al.*, 2009; Zhu and Helliwell, 2011; Hyun *et al.*, 2016; M. Xu *et al.*, 2016*b*). The inverse relationship of miR156 and miR172 abundance in apices of Arabidopsis plants is likely to be part of an intricate gene regulatory network and is recognized by a feedforward loop as AP2 directly binds to *MIR156e* and *MIR172* to induce and repress their expression, respectively (Yant *et al.*, 2010; Jung *et al.*, 2011). In addition, SPL9/SPL15 functionally cooperate with the MADS-box protein SUPPRESSOR OF OVEREXPRESSION OF CONSTANS 1 (SOC1) to activate *FRUITFULL* (*FUL*) and *TARGET OF FLC AND SVP1* (*TFS1*) (Wang *et al.*, 2009; Wu *et al.*, 2009; Hyun *et al.*, 2016; Richter *et al.*, 2019). While SOC1 promotes DNA looping and orchestrates the recruitment of the chromatin remodeller REF6 and BRM to *FUL* and *TFS1*, SPL9/SPL15 stabilize the DNA loop to induce an epigenetic switch through activation of transcription (Hyun *et al.*, 2016; Richter *et al.*, 2019). Bioactive GAs are important for SPL9/SPL15 function as their interaction with the otherwise GA-labile DELLA proteins inhibits SPL9/SPL15 transactivation activity during floral transition (Yu *et al.*, 2012; Hyun *et al.*, 2016). In contrast, the transactivation activity of SPL9 is potentiated through the interaction with DELLA proteins during reproductive development to enhance the expression of the floral meristem identity gene *AP1* (Yamaguchi *et al.*, 2014).

## Phytohormone-dependent floral induction in *Arabidopsis thaliana*

Spatially distinct regulatory roles for bioactive GAs have been suggested in the promotion of flowering under non-inductive SD and inductive LD photoperiodic conditions (Galvão *et al.*, 2012; Porri *et al.*, 2012). TEM genes were shown to link photoperiod and GA pathways by directly binding to and repressing the expression of GA metabolic enzyme genes *GIBBERELLIN 3-OXIDASE1* (*GA3ox1*) and *GA3ox2* (Hu *et al.*, 2008; Yamaguchi, 2008; Osnato *et al.*, 2012). Similarly, the floral repressors *SHORT VEGETATIVE PHASE* (*SVP*) and *FLC* control GA metabolism through the regulation of GA20- and GA2-oxidases (Andrés *et al.*, 2014; Mateos *et al.*, 2015). GA deficiency leads to the stabilization of the otherwise GA-labile DELLA proteins GIBBERELLIC ACID INSENSITIVE (GAI), REPRESSOR OF ga1-3 (RGA), RGA-LIKE1 (RGL1), RGL2, and RGL3 that inhibit transactivation activity of CO through a physical interaction (Schwechheimer, 2011; Wang *et al.*, 2016; F. Xu *et al.*, 2016).

The WRKY-type TFs WRKY71 and WRKY75 activate the expression of *FT* in inductive LD conditions through direct binding to W-boxes located within the promoter of *FT* (Yu *et al.*, 2016; Zhang *et al.*, 2018). The transactivation activity of WRKY75 is inhibited by interactions with DELLA proteins, thus leading to a reduced expression of *FT* (Fig. 2) (Zhang *et al.*, 2018). Similarly, WRKY12 and WRKY13 were also found to interact with DELLAs, and oppositely regulate flowering under non-inductive SD conditions. Interestingly, whereas the expression of *WRKY12* increases as the plant ages to promote flowering, the expression of the floral repressor *WRKY13* concomitantly declines (Li *et al.*, 2016).

Elucidation of GA responses in seedlings revealed that gene expression of virtually all GA-regulated genes depends on the chromatin-remodelling factor PKL (Park *et al.*, 2017). PKL function is inhibited through physical interaction with DELLAs, thus reshaping the epigenetic landscape of its immediate downstream target genes (Zhang *et al.*, 2014; Park *et al.*, 2017). It is noteworthy that the ABA-responsive element (ABRE)-binding factor 3 (ABF3) and ABF4 engage in NF-YC interactions to promote flowering by activating *SOC1* gene expression in the leaf, whereas they delay flowering by repressing *SOC1* transcription in the apex (Riboni *et al.*, 2013, 2016; Hwang *et al.*, 2019). Thus, the spatio-temporal control of *SOC1* gene transcription via ABF3/ABF4 and NF-YC modulates the drought escape response in Arabidopsis. Moreover, the formation of REF6/NF-Y (namely NF-YA–NF-YB–NF-YC) complexes is disrupted through physical interactions between DELLAs and NF-Ys, thus suppressing *SOC1* gene activation and the floral response in Arabidopsis (Hou *et al.*, 2014).

FUL and TCP15, but probably also TCP14, bind to the promoter of *SOC1* to activate its expression downstream from GA (Torti *et al.*, 2012; Balanzà *et al.*, 2014; Lucero *et al.*, 2017). TCP14 and TCP15 also constitute a point of convergence for GA and cytokinin (CK) signalling as both TCPs interact with DELLA proteins and the *O*-fucosyltransferase SPINDLY (SPY), which suppresses GA signalling and promotes CK responses (Steiner *et al.*, 2012; Davière *et al.*, 2014; Zentella *et al.*, 2017). Similarly, the GATA-type TF genes *GATA*, *NITRATE-INDUCIBLE*, *CARBON-METABOLISM INVOLVED* (*GNC*), and *CYTOKININ-RESPONSIVE GATA FACTOR1* (*CGA1*)/*GNC-LIKE* (*GNL*) are downstream factors of GA and CK signalling and involved in a cross-repressive interaction with *SOC1* to regulate floral and greening response (Naito *et al.*, 2007; Richter *et al.*, 2010, 2013). Although GNC and CGA1/GNL were found to interact with the transcriptional co-regulator SNL1 in yeast, which is part of HDAC complexes, both GATAs induce the expression of *SMZ* and *SNZ* to regulate flowering (Bowen *et al.*, 2010; Gras *et al.*, 2018). Interestingly, the transcriptional repressor function of TOE1 and TOE2 is inhibited through interactions with the otherwise jasmonate (JA)-labile JASMONATE-ZIM DOMAIN (JAZ) proteins, thus linking JA signalling to flowering time (Zhai *et al.*, 2015). Furthermore, the JA-activated MYC-type TFs directly bind to a promoter-proximal region in *FT*, further supporting the contribution of JA to the floral response (Wang *et al.*, 2017).

## Conclusion

The mechanism underlying seasonal flowering has been attracting a lot of attention for a long time. Initial genetic studies on Arabidopsis have identified many molecular components that either positively or negatively regulate competence to flower downstream of environmental and endogenous cues. Subsequently, further genetic studies together with genome-wide analyses have revealed the crosstalk between these regulators, illustrating the networks that are progressively increasing in complexity over the last years. One of the most important features in this network is the convergence of the regulatory pathways on the integrator genes. As we introduced, recent studies have demonstrated detailed molecular mechanisms by which different signals are integrated into *FT* expression in leaves. Flowering time control via the vernalization pathway is not explained due to space limitation, but there are a number of articles that review recent findings on the vernalization pathway (Bloomer and Dean, 2017; Xu and Chong, 2018). On the other hand, there is still less information available for the signal integration in the SAM to reorganize its identity upon the arrival of FT protein. Future studies will elucidate such mechanisms more precisely and will deepen our knowledge on developmental plasticity.

## Supplementary data

Supplementary data are available at *JXB* online.

**Table S1.** List of the genes regulating flowering time in Arabidopsis.

**Table S2.** List of the genes that control flowering time (transcription factors).

**Table S3.** List of the genes that regulate the transcript level of transcription factors.

**Table S4.** List of the genes that regulate the function of transcription factors.

**Table S5.** List of the genes that regulate flowering via epigenetic control.

**Table S6.** The rest of the genes for flowering time control.

eraa057_suppl_supplementary_tables_S1_S6
